# Effect of telmisartan, angiotensin‐converting enzyme inhibition, or both, on proteinuria and blood pressure in dogs

**DOI:** 10.1111/jvim.16102

**Published:** 2021-03-26

**Authors:** Brittany L. Fowler, Darko Stefanovski, Rebecka S. Hess, Kathryn McGonigle

**Affiliations:** ^1^ University of Pennsylvania Matthew J. Ryan Veterinary Hospital Philadelphia Pennsylvania USA; ^2^ Present address: Port City Veterinary Referral Hospital Portsmouth, NH

**Keywords:** angiotensin receptor blocker, hypertension, protein losing nephropathy, urine protein to creatinine ratio

## Abstract

**Background:**

The use of telmisartan (TEL), an angiotensin‐receptor blocker, for the control of systemic hypertension and proteinuria in dogs has not been reported extensively in a clinical setting.

**Objectives:**

To determine the effects of an angiotensin‐converting enzyme inhibitor (ACEi) alone, ACEi in combination with TEL, or TEL alone on systolic blood pressure and proteinuria in dogs with protein losing nephropathy (PLN).

**Animals:**

Forty‐two client‐owned dogs being treated for PLN.

**Methods:**

Retrospective observational study of medical records of dogs at a university teaching hospital from 2012 to 2018 with the use of benazepril or enalapril alone, TEL alone, or both modalities for the management of PLN. Noninvasive blood pressure and urine protein to creatinine ratio (UPC) were compared among the treatment groups over time. A multivariable mixed‐effects linear regression model followed by post hoc analysis was used to estimate the marginal means and differences between the treatment groups.

**Results:**

In comparison to group ACEi alone, combination treatment of an ACEi with TEL significantly reduced (*P* = .007) systolic blood pressure by 13 mm Hg (95% confidence interval [95% CI]: 4‐22 mm Hg). Angiotensin‐converting enzyme inhibitor + TEL in comparison to ACEi alone showed significant (*P* = .01) reduction in UPC of 2.5 (95% CI: 0.6‐4.4). The UPC of group ACEi + TEL was significantly lower (*P* = .01) in comparison to TEL alone by 3.8 (95% CI: 0.8‐6.8).

**Conclusions and Clinical Importance:**

Telmisartan can be used to treat systemic hypertension and proteinuria in dogs.

**Abbreviations**
ACEi: angiotensin‐converting enzyme inhibitor
ARB: angiotensin receptor blocker
AT‐II: angiotensin‐II
CKD: chronic kidney disease
PLN: protein losing nephropathy
RAAS: renin‐angiotensin‐aldosterone system
TEL: telmisartan
UPC: urine protein to creatinine ratio

## 1 INTRODUCTION

Proteinuria is a negative prognostic indicator for chronic kidney disease (CKD), and is associated with an increased risk for uremic crisis, progressive worsening of azotemia, and death in dogs.^1^ There is a strong consensus that attention must be given to the detection, evaluation, treatment, and monitoring of proteinuria in dogs to improve outcomes in dogs with CKD and in protein losing nephropathy (PLN) of other origin.^2^ Dogs with CKD can also develop systemic hypertension, which has a multifactorial pathogenesis. In humans, hypertension because of renal disease is attributed to impaired renal sodium handling, excessive activation of the renin‐angiotensin‐aldosterone system (RAAS), sympathetic nervous system hyperactivity, and endothelial factors.^3^

The major target system for medically reducing proteinuria and controlling blood pressure is the RAAS, with the most common medication used in dogs being an angiotensin‐converting enzyme inhibitor (ACEi). However, the production of angiotensin‐II (AT‐II) can occur through non‐ACE pathways, which are left unaffected by ACE inhibition.^4^, ^5^ Telmisartan (TEL) is a selective angiotensin receptor antagonist, and blocks the AT‐II type I receptor with a high affinity.^4^, ^5^ Because TEL acts directly on the AT‐II receptor rather than preventing the production of AT‐II itself, it can block AT‐II independent of the pathway by which it is produced.^5^

In 2013, the first approved angiotensin receptor blocker (ARB), TEL (Semintra, Boehringer Ingelheim Vetmedica GmbH, 4 mg/mL), became available for use in cats with CKD in the European Union (EU).^6^ A large prospective multicenter controlled clinical trial evaluated the effect of TEL on proteinuria in cats compared to benazepril.^7^ The results showed that cats in the TEL group had a significantly decreased urine protein to creatinine ratio (UPC) when compared to baseline, whereas these changes were not observed in the benazepril group.^7^ Prospective placebo‐controlled studies in cats and 1 case report have also demonstrated a significant reduction in systolic blood pressure with TEL compared to placebo.^8^, ^9^, ^10^

The veterinary literature is sparse regarding the use of TEL in dogs. A study evaluating the effect of TEL on renal excretory function in conscious healthy dogs concluded that TEL promoted the excretion of water, sodium, and chloride without influencing potassium or creatinine excretion.^11^ The only clinical report of the utility of TEL in a dog is a case report detailing refractory proteinuria in a middle‐aged Beagle dog, which resolved with the use of TEL.^12^

The paucity of information for the role of TEL in the management of proteinuria and systemic hypertension in dogs led to the main study aims: to describe a study sample of dogs treated for PLN with an ACEi alone, ACEi with the addition of TEL, and with TEL alone; and to determine if there was a significant difference in systolic blood pressure or proteinuria among dogs in these same groups. It was hypothesized that dogs treated with TEL alone or in combination with an ACEi would have a significantly lower systolic blood pressure and UPC compared to those treated with ACEi alone. Lastly, we aimed to assess changes in clinicopathologic variables including potassium, phosphorus, blood urea nitrogen (BUN), and creatinine amongst treatment groups.

## 2 MATERIALS AND METHODS

A retrospective observational study for dogs with PLN was performed. All medical records were initially screened to identify dogs with suspect renal proteinuria. Three study samples were created for evaluation and review. Group ACEi dogs received benazepril or enalapril as the primary medical treatment to reduce proteinuria. Group ACEi + TEL dogs received TEL in addition to benazepril or enalapril. Group TEL dogs received TEL and no ACEi.

Group ACEi served as the control treatment group for the study. Once all TEL cases were identified and reviewed (either group ACEi + TEL or group TEL), additional medical records of dogs prescribed an ACEi alone (benazepril or enalapril) between 2012 and 2018 at the University of Pennsylvania School of Veterinary Medicine for the treatment of PLN were evaluated. The number of unique control treatment group dogs equaled the number of cases identified to be in group ACEi + TEL. Dogs in the ACEi control treatment group were sex, age, and reproductive status matched to dogs in the ACEi + TEL group. When an exact match was not possible, the control treatment group dogs were chosen with the age closest to those in the ACEi + TEL group.

Medical records of all client‐owned dogs prescribed TEL between 2012 and 2018 for the treatment of PLN at the University of Pennsylvania School of Veterinary Medicine were reviewed. Dogs were included if they were treated with TEL, ACEi, or both modalities at the University of Pennsylvania School of Veterinary Medicine, and had a noninvasive systolic blood pressure measurement, a UPC, and clinicopathologic testing, which included BUN, creatinine, potassium, and phosphorus performed before initiating TEL. The inclusion criteria also required at least 1 follow‐up appointment after treatment began, which included a complete blood count and chemistry panel (Vitros 4600 Chemistry System, Ortho‐Clinical Diagnostics, Rochester, New York), systolic blood pressure measurement (Dispomed Doppler Medical Electronics 811‐B, Turnersville, New Jersey), and UPC (Vitros 4600 Chemistry System, Ortho‐Clinical Diagnostic). There was no requirement for the number of days after beginning the treatment for the first recheck to occur, and the time between rechecks was variable based on the clinician discretion and owner compliance. Follow‐up testing was included whether performed at the University of Pennsylvania School of Veterinary Medicine or with the dog's primary care veterinarian. Dogs were excluded if they were not prescribed TEL by the University of Pennsylvania School of Veterinary Medicine, did not have a blood pressure measurement recorded, UPC performed, bloodwork (BUN, creatinine, potassium, and phosphorus) available for review, or if at the time of follow‐up an acute kidney injury was associated with an infectious etiology. Dogs with concurrent diseases known to cause proteinuria, systemic hypertension, or both proteinuria and hypertension, or receiving medications known to affect blood pressure and proteinuria were not excluded from the study sample. Dogs dropping out because of death or being lost to follow‐up were to be expected, and data from these dogs were included up until the point at which their last recheck was performed.

The baseline visit was defined as the time that an ACEi or TEL were first administered to a dog. Data recorded included signalment, reproductive status, current medication and dosing (milligrams/kg/day) of ACEi (benazepril or enalapril), TEL, or both ACEi and TEL when used together, time point in days from baseline visit, serum BUN, creatinine, potassium and phosphorus, systolic blood pressure, and UPC. These values were recorded at each time point available for up to 5 rechecks after beginning treatment with the medication of interest. For the purpose of this study, a rise in potassium was defined as an increase in serum potassium by ≥0.5 mEq/L at any given recheck. This value was based on available human literature evaluating significant change in potassium with the use of ACEi and ARBs.^13^, ^14^ Normal systolic blood pressure was defined as 100 to 150 mm Hg and normal UPC was defined as <0.20.

Medical management changed over time in some dogs with the addition or removal of 1 of the medications of interest. Thus, single dogs could be included in multiple treatment groups if the medical record included baseline information before switching groups, and recheck information after the medication protocol was changed. Because of the nature of the study, a washout period was not performed when transitioning dogs from 1 group to the next.

### 2.1 Statistical analyses

All analyses were conducted with 2‐sided tests of hypotheses and a *P*‐value <.05 as the criterion for statistical significance (Stata 15MP, StataCorp, State College, Texas). Descriptive analyses included computation of medians, minimum, and maximum for continuous variables and tabulation of categorical variables. Tests of normal distribution (Shapiro‐Wilk tests for normality) were performed to determine the extent of skewness. Frequency counts and percentages were used for summarizing categorical variables (eg, sex, signalment and others). The number of dogs within each group changed with each follow‐up time point as some dogs moved between groups. In addition, some dogs were removed from the study as they were lost to follow‐up, could no longer tolerate the medication, or were euthanized.

Inference statistical analysis was conducted in 2 steps. First, univariate linear regression was used to identify statistically significant confounders associated (*P* < .20) with the main outcomes (BUN, creatinine, potassium, phosphorus, blood pressure, and UPC).^15^ Variables examined as possible confounders were age, sex, and reproductive status. Second and final, a multivariable mixed‐effects linear regression model was used that included the fixed effects of treatment and the interaction between treatment and observation sample as categorical variables and any confounders identified in the previous steps. Mixed‐effects linear regression was chosen because of large numbers of missing values in the dataset, subjects moving from 1 group to another, repeated measures over time, and departures from normality of the outcome. The random effects were set on the level of the individual animal. Robust estimation of the variance was used to adjust for any possible departures from the normality of the outcome. Post hoc analysis was used to estimate the marginal means and 95% confidence intervals (95% CIs) and differences between the treatment groups. Least Significant Difference method was used to adjust for multiple comparisons. When using statistical models with 2 or more independent variables (fixed or random‐effects or any statistical interaction between them) to explain a given outcome (dependent variable), the marginal means for a given outcome in respect to 1 of the independent variables are the means of the dependent variable averaged across all levels of the other independent variables.^16^

## 3 RESULTS

### 3.1 Study demographics

All dogs prescribed TEL at the University of Pennsylvania School of Veterinary Medicine between 2012 and 2018 were considered for evaluation, and 42 individual dogs were ultimately included within the study. Out of all the dogs started on TEL within the study window at the University of Pennsylvania School of Veterinary Medicine, only 2 were excluded as 1 owner never started the medication, and another pet was euthanized before any rechecks.

The number of dogs in each treatment group at different time points is recorded in Table 1. Median age of dogs in the TEL group (11.8 years; range, 6‐15 years) was significantly higher than the median age of dogs in the ACEi and ACEi + TEL groups (10 years; range, 2‐13 years and 10 years; range, 1.5‐15 years, respectively; *P* < .001 for each). There was no difference in age between the ACEi and ACEi + TEL groups. Sex and reproductive status at each time point are reported in the Supplementary Table S1. There was no difference in sex distribution amongst dogs in the 3 treatment groups. Diagnosed comorbidities included: diabetes mellitus (2), chronic hepatitis (2), myxomatous mitral valve disease (4), urinary cystolithiasis (2), hyperadrenocorticism (2), polycythemia vera (2), chronic enteropathy (2), glaucoma (1), atopic dermatitis (1), neoplasia (spindle cell tumor [1], mast cell tumor [1], hematoma [1], hepatocellular carcinoma [1]), collapsing trachea (1), bone‐marrow targeting immune‐mediated anemia (1), and arrhythmogenic right ventricular cardiomyopathy (1). One dog with hyperadrenocorticism was in group ACE + TEL and the other dog was in group TEL.

**TABLE 1:** Number of dogs with protein losing nephropathy (PLN) in each treatment group and time from enrollment by treatment group and visit number

Visit number	ACEi^a^		ACEi + TEL^b^		TEL^c^	
	Median days from enrollment	Number of cases	Median days from enrollment	Number of cases	Median days from enrollment	Number of cases
1	0	20	0	19	0	3
2	14 (1‐128)	20	29 (7‐142)	19	10 (8‐56)	3
3	36 (12‐129)	18	63.5 (14‐162)	19	36 (11‐126)	5
4	74 (26‐395)	13	124.5 (55‐230)	15	62 (12‐312)	10
5	104 (65‐223)	6	145 (91‐446)	8	97 (32‐223)	7
6	133 (122‐203)	3	337 (179‐495)	2	162 (101‐315)	4

^a^ ACEi, dogs with PLN treated with ACE inhibitor alone.

^b^ ACEi + TEL, dogs with PLN treated with ACE inhibitor and telmisartan.

^c^ TEL, dogs with PLN treated with telmisartan alone.

Concurrent medications included amlodipine (22), insulin (2), trilostane (2), aspirin (11), clopidogrel (17), prednisone (4), omega 3‐fish oils (23), aluminum hydroxide (8), omeprazole (13), mycophenolate (7), Denamarin (5), ursodiol (3), silymarin (2), phenobarbital (1), sodium polystyrene (2), and hydroxyurea (2).

Out of the 42 individual dogs within the study, 37 of them had all their examinations and rechecks performed at the University of Pennsylvania School of Veterinary Medicine. Out of the 5 dogs that had 1 or more follow‐up examinations performed with their primary care veterinarian, 2 dogs had 1 exam performed with their primary care veterinarian and 5 visits at the University of Pennsylvania School of Veterinary Medicine. The other 3 dogs had 2 exams at their primary care veterinarian.

Out of the 42 individual dogs within the study, 13 dogs were represented within 2 or more groups. Three dogs were transitioned from group ACEi to group ACEi + TEL, and this transition occurred after visit 3 or 4. Five dogs were transitioned from group ACEi to group TEL and this transition occurred after visit 2 to visit 4. Two dogs transitioned from group ACEi + TEL to group TEL, after visits 2 and 3. One dog was transitioned from group ACEi + TEL to group ACEi at visit 5. One dog was represented in all 3 groups, and 1 dog started in group ACEi + TEL, was transitioned to group TEL, and shortly after transitioned back to group ACEi + TEL.

At entry into the study, 31 dogs were identified to have hypertension (systolic blood pressure >150 mm Hg), and 22 of those dogs were subsequently treated with amlodipine. Out of the dogs with hypertension where amlodipine was not used the median blood pressure was 170 mm Hg (range, 155‐226 mm Hg).

### 3.2 Drug doses

The median dose of enalapril or benazepril used for dogs in the ACEi group was 0.68 mg/kg/day (range, 0.25‐1.96 mg/kg/day; interquartile range, 0.54), whereas the median dose of enalapril or benazepril used for dogs in the ACEi + TEL group was 1.75 mg/kg/day (range, 0.22‐2.3 mg/kg/day; interquartile range, 0.82). The median dose of TEL in dogs in the ACEi + TEL group was 0.93 mg/kg/day (range, 0.25‐1.8 mg/kg/day; interquartile range, 0.48) and in the TEL group it was 0.9 mg/kg/day (range, 0.19‐1.94 mg/kg/day; interquartile range, 0.17).

### 3.3 Telmisartan effects on serum variables of interest

Table 2 reports the marginal means and 95% CI of each variable of interest, including BUN, creatinine, phosphorus, potassium, blood pressure, and UPC by treatment group over time. The marginal mean BUN in the TEL group (Table 2) was significantly higher (*P* = .04) compared to the marginal mean BUN in the ACEi group by 16 mg/dL (95% CI: 0.8‐29.8 mg/dL). There was no significant difference in BUN between the ACEi group and the ACEi + TEL group, or between the TEL group and the ACEi + TEL group. There was no significant difference in marginal mean creatinine or phosphorus over time between any of the groups.

**TABLE 2:** Marginal means and 95% confidence interval (95% CI) of serum blood urea nitrogen (BUN), serum creatinine, serum phosphorus, serum potassium, blood pressure, and urine protein to creatinine ratio (UPC) by treatment group over time

Parameter measured	ACEi^a^		ACEi + TEL^b^		TEL^c^	
	Marginal mean	95% CI	Marginal mean	95% CI	Marginal mean	95% CI
BUN (mg/dL)	37	28‐45	46	37‐54	53	40‐66
Creatinine (mg/dL)	1.9	1.5‐2.6	1.8	1.4‐2.1	1.5	1.2‐1.9
Phosphorus (mg/dL)	5.2	4.7‐5.7	5.8	5‐6.6	5.9	4.8‐7.1
Potassium (mEq/L)	4.9	4.7‐5.1	5.1	5‐5.3	5.1	4.9‐5.3
Systolic blood pressure (mm Hg)	158	152‐164	145	139‐153	147	126‐168
UPC	6.3	4.6‐8	3.8	2.3‐5.2	7.6	4.9‐10.2

*Note:* Normal systolic blood pressure was defined as 100 to 150 mm Hg. Normal urine protein to creatinine ratio was defined as <0.20.

^a^ ACEi, dogs with PLN treated with ACE inhibitor alone.

^b^ ACEi + TEL, dogs with PLN treated with ACE inhibitor and telmisartan.

^c^ TEL, dogs with PLN treated with telmisartan alone.

The univariate analysis showed that potassium concentration was dependent on age, whereas there was no effect of sex or reproductive status on potassium concentration. This was adjusted for in the mixed‐effects linear regression model. A rise in potassium was identified in 46.3% of dogs in the ACEi group, 51.2% of dogs in the ACEi + TEL group, and 28% of dogs in the TEL group. The marginal mean serum potassium over time was higher in the ACEi + TEL group (Table 2) compared to the ACEi group by 0.2 mEq/L (95% CI: 0.04‐0.4 mEq/L, *P* = .02). There was no significant difference in serum potassium between the ACEi + TEL group and the TEL group, or between the ACEi group and the TEL group. Treatment was discontinued because of the serum potassium (6.2 mEq/L) in only 1 dog from the ACEi + TEL treatment group.

### 3.4 Telmisartan effect on blood pressure and proteinuria

Besides the time of observation, the univariate analysis showed that systolic blood pressure was dependent on reproductive status, whereas there was no effect of sex or age on blood pressure. Hence, the mixed‐effects linear regression model included this significant confounder. When analyzing all dogs within each group over time, the marginal mean systolic blood pressure in the ACEi + TEL group (Table 2) was significantly lower (*P* = .007) than the marginal mean blood pressure in the ACEi group by 13 mm Hg (95% CI: 4‐22 mm Hg). The marginal mean systolic blood pressure in the TEL group was lower than in the ACEi group, but did not reach significance (*P* = .31; Table 2). There was no difference in marginal mean systolic blood pressure between the TEL group and the ACEi + TEL group (Table 2).

When adjusted for time, the marginal mean UPC was significantly lower in the ACEi + TEL group (*P* = .01; Table 2) compared to the ACEi group by 2.5 (95% CI: 0.6‐4.4). There was no difference in the marginal mean UPC of the TEL group and the ACEi group (*P* = .40; Table 2). The TEL group had a significantly higher (*P* = .01) marginal mean UPC over time (Table 2) compared to the ACEi + TEL group by 3.8 (95% CI: 0.8‐6.8).

## 4 DISCUSSION

This study describes and compares a sample of dogs medically treated for proteinuria with an ACEi, TEL, or a combination of ACEi and TEL over time. Findings show that the marginal mean systolic blood pressure is significantly lower in dogs treated with both an ACEi and TEL compared to dogs treated with an ACEi alone. This finding suggests that TEL can be considered for treatment of hypertension in dogs. This study also found that the UPC was lower in dogs receiving combination treatment with an ACEi and TEL compared to ACEi alone. These data support the continued use of TEL in the management of proteinuria in dogs.

Looking further at the effects of TEL use and proteinuria, the marginal mean UPC was significantly higher for dogs in the TEL group over time compared to the marginal mean UPC for dogs in the ACEi + TEL group over time. This unexpected finding might be explained with the consideration that these dogs had more advanced disease. In current clinical settings, many clinicians do not prescribe TEL for proteinuria treatment unless the dogs have developed progressive proteinuria in the face of ACEi treatment or experienced an adverse effect prohibiting future use (ie, acute kidney injury, a rise in potassium, gastrointestinal adverse effects). Based on current practices, it is most likely that dogs transitioned to the TEL group had higher starting UPC measurements than dogs in the ACEi group and the ACEi + TEL group. Therefore, based on our statistical evaluation of marginal means at the group level, the comparison reflects that the overall marginal mean was higher for dogs in group TEL compared to group ACEi + TEL. It is also possible that the ACEi + TEL combination is a superior form of treatment for proteinuria than TEL alone. Future prospective studies should be performed with dogs that are matched for starting UPC measurements and randomly allocated to receive each treatment. These treatment groups can be analyzed at the individual level, and considerations can also be made to assess the percent decrease in UPC over time.

When evaluating the effects of TEL on blood pressure, the results of this study suggest an additive effect of ACE inhibition and TEL. Telmisartan is an approved drug to treat systemic hypertension in cats, and it is important to be aware of this effect when using TEL to treat proteinuria in dogs. Further prospective studies are warranted to evaluate the effect of TEL alone or in combination treatment on blood pressure in dogs.

Although it did not reach significance, the blood pressure was higher for dogs in the TEL group compared to the ACEi + TEL group. This could be reflective of more advanced disease in dogs treated with TEL alone. In addition, many dogs were added to the TEL group or transitioned into the TEL group toward the end of the study window and had fewer visits to evaluate. The median number of days from enrollment for dogs in the TEL group was smaller compared to the median number of days from enrollment for dogs in the ACEi + TEL group. It is possible that the blood pressure would have continued to decrease with more time after implementation of the TEL treatment as a sole agent.

One concern with the use of TEL in dogs is the risk for hyperkalemia. Multiple human studies have been performed evaluating the hyperkalemic risk in hospitalized humans with various ARBs.^13^, ^14^ The results indicated that humans frequently displayed a rise in serum potassium with ARB treatment; however, clinically significant hyperkalemic events were rare.^13^ Another human study evaluated the prevalence of hyperkalemia with ACEi treatment compared to ARB treatment.^14^ This study showed that severe hyperkalemia (defined as >6 mEq/L) was only observed in 1% to 3% of people and there was a higher prevalence in ARBs compared to ACEi treated people.^14^ It was postulated that this could be because of the fact that the study sample treated with ARBs had a higher prevalence of congestive heart failure and was already on potassium sparing diuretics, potassium replacement treatment, or both. The current study showed that some dogs' potassium increased throughout the study with the use of these medications (a rise in potassium was defined as an increase in serum potassium by ≥0.5 mEq/L), although the lowest frequency of a potassium rise was seen with the dogs treated with TEL alone. Despite an increasing potassium, only 1 clinician recommended discontinuation of medications (benazepril and TEL) because of the serum potassium concentration. This suggests that the degree of serum potassium change was not clinically relevant in most dogs.

Several limitations are noted in this exploratory study because of the retrospective study design. Equal numbers of dogs to represent each treatment group were not available. Second, a limited number of follow‐up visits were available for some dogs because of transition to other treatment groups, and the recheck appointments were performed over a wide range of days after initiating treatment or performing medication adjustments. Dog group transitions occurred because of inadequate response to treatment, adverse effects, and clinician preference. Third, there were different managing clinicians with no standard treatment protocol for dosing or time of transition from 1 medication to another. When dogs did change treatment groups, no washout period was performed before instituting the new treatment.

The lack of standardization regarding drug dose is important to note when looking at the median dose utilized for both ACEi and TEL, as well as the dose ranges. The current consensus statement for treatment of glomerular disease in dogs recommends minimum ACEi dosing of 0.5 mg/kg/day up to a maximum of 2 mg/kg/day.^17^ Dogs in group ACEi received a median dose of 0.68 mg/kg/day compared to group ACEI + TEL dogs that received a median dose of 1.75 mg/kg/day. This might indicate that dogs treated with ACEi alone had mild proteinuria as it was treated with the low end of the dosing interval, compared to dogs on combination treatment where the median dose was close to the maximum dose per day. The dose ranges also show that some dogs in both groups were receiving subtherapeutic doses of ACEi that might have impacted the success of proteinuria reduction. Additional studies could be performed to determine if these subtherapeutic doses were utilized because of dog drug tolerance, owner noncompliance, or clinician preference. Given the lack of studies regarding TEL use in dogs for proteinuria, there is no true consensus regarding dose. The 2013 consensus statement for glomerular disease in dogs discusses a dose of 1 mg/kg once daily; however, this recommendation was based off 1 unpublished observation looking at TEL in normal dogs at 1 mg/kg/day compared to enalapril at 0.5 mg/kg twice daily.^17^ The product Semintra is approved to treat proteinuria in cats in the EU at 1 mg/kg once daily, which likely explains the median dose utilized in this study sample of dogs (0.93 mg/kg/day group ACEI + TEL; 0.9 mg/kg/day group TEL). However, some dogs treated with TEL received doses as low as 0.19 mg/kg/day, which likely played a role in the effectiveness in treatment. Additional studies should be performed to determine if the success at proteinuria reduction is dose dependent.

An additional limitation included that some of the follow‐up appointments were completed with the primary care veterinarian, using different chemistry analyzer equipment with different reference ranges than the University of Pennsylvania School of Veterinary Medicine. Given that 37/42 dogs had all examinations and lab work performed at the University of Pennsylvania School of Veterinary Medicine, we suspect that the few rechecks performed at a particular dog's primary care veterinary office did not significantly affect the results found in this study. Dietary management was also not included in the analysis of this study for 2 main reasons; (a) the medical records did not consistently report the diet the dog was consuming and (b) many owners were reported to be giving table food and multiple diets together to entice the dogs to eat, making true analysis of 1 diet impossible.

Another consideration when reviewing the study results is that several dogs had concurrent diseases and were receiving additional medications which could have affected blood pressure, clinicopathologic variables, and confounded treatment decisions regarding ACEi or TEL use (amlodipine, 22; prednisone, 4; trilostane, 2; omega‐3 fatty acids, 23). Given that both dogs with Cushing's disease were treated with trilostane, the contribution of proteinuria from hyperadrenocorticism should be minimal. Out of the 4 dogs treated with prednisone, 3 of them were short tapering courses, decreasing the likelihood for any significant lasting impact on proteinuria. Lastly, the sample size was small, dictated by the total use of TEL at the University of Pennsylvania School of Veterinary Medicine within the study window.

## CONFLICT OF INTEREST DECLARATION

The authors declared no potential conflicts of interest.

## OFF‐LABEL ANTIMICROBIAL DECLARATION

Authors declare no off‐label use of antimicrobials.

## INSTITUTIONAL ANIMAL CARE AND USE COMMITTEE (IACUC) OR OTHER APPROVAL DECLARATION

Authors declare no IACUC or other approval was needed.

## HUMAN ETHICS APPROVAL DECLARATION

Authors declare human ethics approval was not needed for this study.

## Supporting information


**Appendix** **S1**: Supporting Information
