# Supplementary material for: Effect of telmisartan, angiotensin‐converting enzyme inhibition, or both, on proteinuria and blood pressure in dogs
Source: J Vet Intern Med. 2021 Mar 26;35(3):1231–7. doi: 10.1111/jvim.16102 (PMC8163128; doi:10.1111/jvim.16102)
Supplement: Supplementary file 1 — Appendix S1: Supporting Information [file JVIM-35-1231-s001.pdf]

Table S1. Sex and reproductive status by treatment group and visit number.

Visit Number	ACEi*				ACEi + TEL**				TEL***			
	IF	SF	IM	CM	IF	SF	IM	CM	IF	SF	IM	CM
**1**	0	15	2	3	2	9	4	4	0	1	0	2
**2**	0	15	2	3	2	9	4	4	0	1	0	2
**3**	0	13	2	3	2	10	4	3	0	2	0	3
**4**	0	8	2	3	2	7	4	2	0	6	0	4
**5**	1	3	1	1	0	4	2	2	0	5	0	2
**6**	0	2	0	1	0	2	0	0	0	2	1	1

*ACEi, Dogs with protein losing nephropathy (PLN) treated with ACE inhibitor alone

**ACEi + TEL, Dogs with PLN treated with ACE inhibitor and telmisartan

***TEL, Dogs with PLN treated with telmisartan alone

Females are indicated as spayed or intact (SF, IF) respectively. Males are indicated as castrated or intact (CM, IM) respectively.
